# Whole Genome Sequencing of Clinical *Mycobacterium bovis* BCG in Disseminated Infection with Mycotic Aneurysm and ARDS After Intravesical Therapy: A Case Report

**DOI:** 10.3390/ijms27010238

**Published:** 2025-12-25

**Authors:** Yuta Nakagawa, Chie Yamamoto, Hidetake Kawajiri, Makoto Watanabe, Tomomi Yamada, Yukiji Yamada, Daisuke Kambayashi, Keitaro Furukawa, Ryosuke Hamashima, Tetsuhiro Yamano, Tohru Inaba, Kunihiko Kooguchi, Shinichiro Oda, Satoshi Mitarai, Yoko Nukui

**Affiliations:** 1 Department of Infection Control and Laboratory Medicine, Kyoto Prefectural University of Medicine, Kyoto 602-8566, Japan; c111061@koto.kpu-m.ac.jp (Y.N.); chiepon@koto.kpu-m.ac.jp (C.Y.); k-furu@koto.kpu-m.ac.jp (K.F.); hamashim@koto.kpu-m.ac.jp (R.H.); tyamano@koto.kpu-m.ac.jp (T.Y.); inaba178@koto.kpu-m.ac.jp (T.I.); 2 Department of Respiratory Medicine, Japanese Red Cross Kyoto Daini Hospital, Kyoto 602-8026, Japan; 3 Division of Cardiovascular and Paediatric Cardiovascular Surgery, Department of Surgery, Kyoto Prefectural University of Medicine, Kyoto 602-8566, Japan; kawajiri@koto.kpu-m.ac.jp (H.K.);; 4 Department of Emergency Medicine, Kyoto Prefectural University of Medicine, Kyoto 602-8566, Japan; m-wtnb@koto.kpu-m.ac.jp; 5 Department of Clinical Laboratory, Kyoto Prefectural University of Medicine, Kyoto 602-8566, Japan; tomomiy@koto.kpu-m.ac.jp (T.Y.); poohsan@koto.kpu-m.ac.jp (Y.Y.); 6 Cardiovascular Department, Fukuchiyama City Hospital, Fukuchiyama 620-0056, Japan; d-kansuke@io.ocn.ne.jp; 7 Department of Critical Care Medicine, University Hospital, Kyoto Prefectural University of Medicine, Kyoto 602-8566, Japan; koochan@koto.kpu-m.ac.jp; 8 Department of Mycobacterium Reference and Research, Research Institute of Tuberculosis, Japan Anti-Tuberculosis Association, Tokyo 204-8533, Japan; mitarai@jata.or.jp

**Keywords:** intravesical Bacillus Calmette–Guérin, *Mycobacterium bovis*, mycotic aneurysm, acute respiratory distress syndrome, disseminated infection, whole-genome sequencing

## Abstract

Intravesical Bacillus Calmette–Guérin (iBCG) immunotherapy is the standard adjuvant treatment of non-muscle-invasive bladder cancer (NMIBC). Among the potential complications, cases of mycotic aneurysms and acute respiratory distress syndrome (ARDS) are rare but can be life-threatening. Because prior reports have not included whole-genome sequencing (WGS) of clinical *Mycobacterium bovis* BCG (*M. bovis* BCG) isolates to assess whether the infecting strain acquires mutations in vivo, we performed WGS in a severe disseminated iBCG-related infection. A 72-year-old man with bladder cancer underwent iBCG instillation. Twelve months after the final instillation, the patient developed an abdominal aortic aneurysm, which was detected and treated with endovascular aneurysm repair (EVAR). Two months later, the patient presented with fever, abdominal pain, and septic shock. Contrast-enhanced computed tomography (CT) and ^18^F-fluorodeoxyglucose positron emission tomography/CT (FDG-PET/CT) showed rapid aneurysm enlargement. Ziehl–Neelsen staining and PCR of aortic material identified *M. bovis* BCG. Direct PCR on BAL fluid and urine was negative; however, BAL and urine culture subsequently grew *M. bovis* BCG, and PCR performed on the culture isolate confirmed *M. bovis* BCG. Despite combined antituberculosis triplet therapy (isoniazid, rifampicin, and ethambutol), the patient developed ARDS, which gradually improved after surgical management. WGS (with >96% genome coverage) showed the isolate was highly concordant with the vaccine strain and lacked additional virulence-associated mutations, including in *esxM*. This case illustrates that severe systemic iBCG-related complications can occur without detectable in vivo acquisition of virulence-enhancing mutations; however, interpretation is limited by the single-case design and the absence of host genetic susceptibility testing. Our findings underscore the need for prolonged vigilance regarding late-onset vascular and pulmonary complications after iBCG, and highlight the importance of early multidisciplinary management.

## 1. Introduction

Bacillus Calmette–Guérin (BCG) is an attenuated strain of *Mycobacterium bovis* (*M. bovis*), a member of the *Mycobacterium tuberculosis* complex (MTBC). It is used both as a vaccine to prevent severe forms of tuberculosis—with the strongest evidence of protection in infants and young children, particularly against disseminated tuberculosis [1]—and, when administered intravesically, as adjuvant immunotherapy for intermediate- and high-risk non-muscle-invasive bladder cancer (NMIBC) after transurethral resection of bladder tumors [2,3]. Because BCG is derived from *M. bovis*, intravesical BCG therapy represents a human model of mycobacterial host–pathogen interaction and antitumor immunity. Severe systemic complications, therefore, provide an opportunity to examine the interplay between mycobacterial genomic features, host immune responses, and vascular pathology. Intravesical BCG (iBCG) triggers an intense local immune reaction, mainly mediated by cytotoxic T cells and macrophages, which reduces tumor recurrence and progression rates [4,5,6], but also underlies local and systemic adverse events. Rare infectious complications, including disseminated BCG infection, are reported particularly in patients with risk factors such as immunosuppression [7]; in this manuscript, “disseminated BCG infection” refers to systemic BCG disease with evidence of spread beyond the genitourinary tract (e.g., microbiologic detection of BCG in blood and/or in a distant organ or tissue consistent with hematogenous dissemination).

Because BCG is a live organism, it can rarely cause a clinically significant infection [8,9]. Adverse events (AEs) are generally classified as short-term, self-limiting reactions (iBCG-ST/SLAEs) or local/systemic complications (iBCG-L/SCs) [10]. While most patients experience short-term, self-limited irritative symptoms, the incidence of severe iBCG-L/SCs requiring medical treatment or hospitalization, including aneurysm and lung disease, is low (2.35%) [10], may occur from hours to years after the last instillation [10], and can be fatal [10]. Notably, diagnosis across reported severe cases has relied on variable-yield mycobacterial cultures/acid-fast bacilli (AFB) microscopy and nucleic acid amplification tests (NAATs), including polymerase chain reaction (PCR)-based assays; PCR-based methods have higher diagnostic yield, and culture/AFB smear detection is suboptimal in iBCG contexts [10]. Practical guidance on recognition and management of iBCG-L/SCs has therefore emphasized early suspicion, timely microbiological work-up, and escalation to targeted antimycobacterial therapy and/or source control [11].

Clinically significant systemic BCG infection after iBCG is considered more likely when systemic absorption is facilitated (e.g., traumatic catheterization) and/or when host susceptibility is present; nevertheless, disseminated and life-threatening complications have also been reported in patients without overt immunodeficiency, underscoring the need for a high index of suspicion whenever compatible syndromes arise after iBCG exposure [9,11].

Among rare systemic complications, vascular involvement is particularly consequential. Reported presentations include aortitis and mycotic aneurysm/pseudoaneurysm—most commonly involving the abdominal aorta—and may occur in the setting of atherosclerotic disease and/or vascular graft material [12,13]. Pulmonary involvement is also well recognized in disseminated BCG infection (e.g., pneumonitis), whereas acute respiratory distress syndrome (ARDS) appears exceptionally rare yet life-threatening when it occurs [14,15].

Mechanistically, severe phenotypes can be conceptualized as joint effects of (i) pathogen determinants (including substrain background and genetic variation), (ii) host susceptibility (immune status and inflammatory milieu), and (iii) tissue context (e.g., atherosclerotic lesions or prosthetic material) that may facilitate persistence, seeding, and destructive inflammation in vascular and pulmonary beds [12,13,16,17]. BCG substrains exhibit genomic diversity that could plausibly influence clinical phenotypes [16,18], and whole-genome sequencing (WGS) has been applied in selected iBCG-associated infections as a high-resolution approach to confirm BCG and support clinical decision-making [17].

Mutations in genes such as *esxM*, an effector of the type VII secretion system, have been reported to affect macrophage motility and increase the risk of mycobacterial dissemination [19]. However, it remains unclear whether in vivo evolution of BCG contributes to exceptionally severe systemic complications after intravesical therapy.

Here, we report a case of delayed onset mycotic aneurysm followed by disseminated *M. bovis* BCG infection complicated by septic shock and ARDS. Given this unusually severe presentation, we performed WGS on clinical isolates to investigate whether in vivo–acquired virulence-associated mutations, such as *esxM,* might plausibly contribute to dissemination and severity [19].

## 2. Case Presentation

A 72-year-old man presented with fever and abdominal pain. He had no history of diabetes mellitus, chronic kidney disease, chronic liver disease, or autoimmune disease. In March 2023, he was diagnosed with bladder cancer and underwent transurethral resection of the bladder tumor (TURBT). He subsequently started intravesical BCG immunotherapy (Immunobladder^®^, Tokyo-172, 80 mg, Japan BCG Laboratory, Kiyose, Tokyo, Japan) in April–May 2023 with weekly instillations; however, the treatment was discontinued after three completed instillations because drug-induced liver injury developed at the time of the fourth scheduled instillation. Random bladder biopsies in July 2023 showed no evidence of recurrence, and he was thereafter followed without further intravesical therapy. During a routine medical checkup in March 2024, abdominal ultrasonography identified an irregular abdominal aortic aneurysm that had not been present on prior screening ultrasonography in 2023; therefore, it was considered newly detected, and he was referred to our hospital in April 2024 for further evaluation. Contrast-enhanced computed tomography (CT) demonstrated an infrarenal abdominal aortic aneurysm with a partially saccular configuration, irregular mural changes, and increased periaortic fat attenuation, raising concern for an infectious aneurysm in addition to a degenerative etiology. In the same month, he was also evaluated for suspected cholangitis and was treated with endoscopic retrograde cholangiopancreatography (ERCP) and antibiotics, with subsequent symptom resolution (Figure S1).

**Figure 1:**
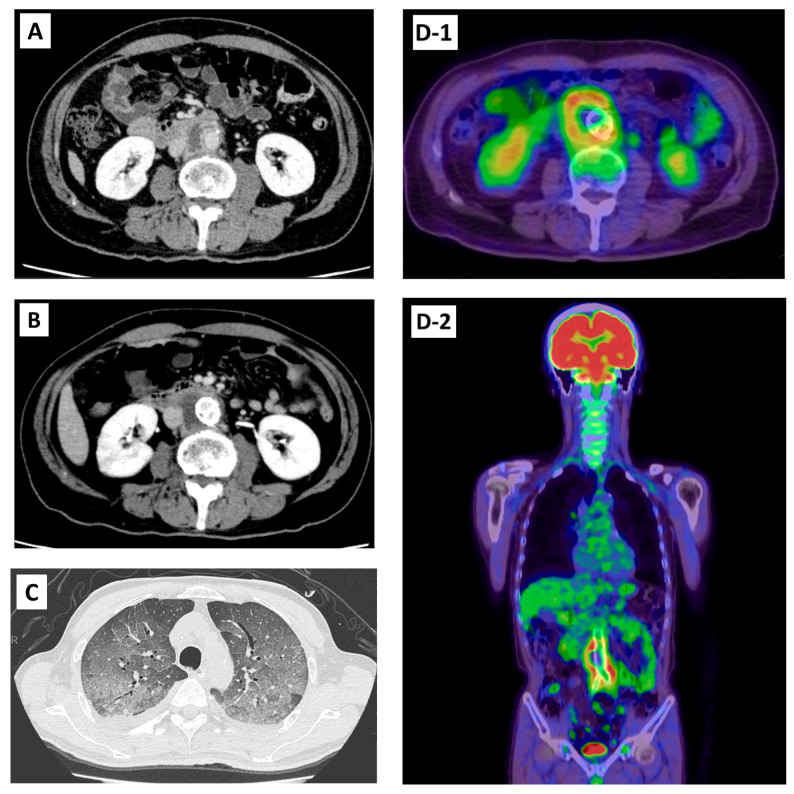
Radiological findings of the abdominal aortic aneurysm and lung involvement. (**A**) Contrast-enhanced computed tomography (CT) in April 2024 showing an abdominal aortic aneurysm without obvious periaortic soft tissue enhancement; (**B**) Contrast-enhanced CT in August 2024 demonstrating rapid enlargement of the abdominal aortic aneurysm with increased enhancement and thickening of the surrounding soft tissue; (**C**) Chest CT in August 2024 showing diffuse bilateral ground-glass opacities (GGOs) in the lungs. (**D**) ^18^F-fluorodeoxyglucose positron emission tomography/CT (FDG-PET/CT) performed in August 2024 demonstrating intense FDG uptake along the aneurysmal aortic wall. (**D-1**) Axial (transverse) fused FDG-PET/CT image showing intense FDG uptake along the aneurysmal aortic wall. (**D-2**) Coronal fused FDG-PET/CT image likewise showing intense FDG uptake along the aneurysmal aortic wall.

**Figure 2:**
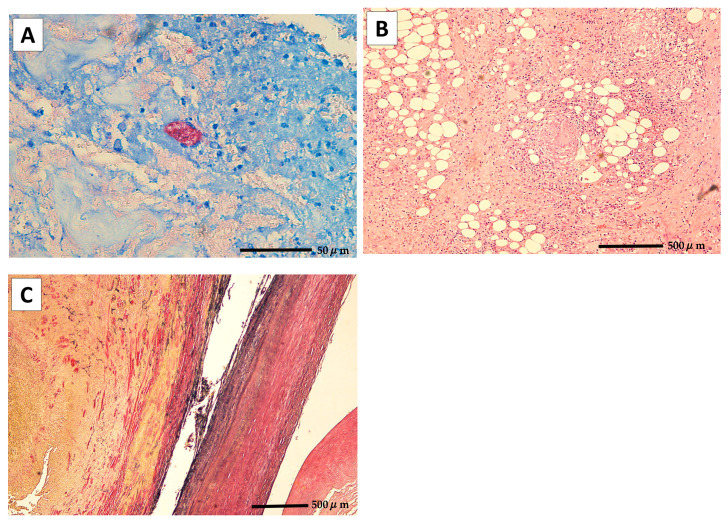
Histopathologic findings of the surgically removed abdominal aortic wall. (**A**) Ziehl–Neelsen stain showing numerous acid-fast bacilli within the aortic wall. Scale bar = 50 μm; (**B**) Hematoxylin and eosin (H&E) section demonstrating dense inflammatory cell infiltrates composed mainly of lymphocytes and plasma cells, with epithelioid cell granulomas and caseating necrosis. Scale bar = 500 μm; (**C**) Elastic van Gieson (EVG) showing disruption and rupture of elastic fibers in the aortic media. Scale bar = 500 μm.

Because he had no systemic symptoms (e.g., fever) and blood cultures for common bacteria and acid-fast organisms obtained on 22 April 2024 were negative, the likelihood of an infectious aneurysm was considered low at that time, and endovascular aneurysm repair (EVAR) was performed on 29 May 2024 with standard perioperative prophylaxis using cefazolin. A GORE EXCLUDER endograft system (W. L. Gore and Associates, Flagstaff, AZ, USA) was deployed, consisting of an aortic extender (GORE EXCLUDER Aortic Extender) and bilateral iliac limbs (ipsilateral: EXCLUDER C3; contralateral: EXCLUDER). In early July 2024, he developed low-grade fever and abdominal pain, which gradually worsened and led to emergency hospitalization in mid-August (day 0). On admission, he was alert and oriented, weighed 57.7 kg, with a body temperature of 38.1 °C, heart rate 98 beats/min, blood pressure 84/50 mmHg, oxygen saturation (SpO_2_) 88% on room air, and respiratory rate 24 breaths/min. Tenderness was noted in the midline of the abdomen and the right quadrant. Laboratory tests revealed liver and renal dysfunction, and elevated inflammatory markers (Table 1). Because recurrent cholangitis was suspected, he underwent repeat ERCP and received antibiotic therapy; therefore, the planned aortic surgery was postponed. The interferon-gamma release assay (IGRA; T-SPOT.TB^®^, Revvity Japan Co., Ltd., Yokohama, Japan) was negative; however, this result does not exclude *M. bovis* BCG infection because IGRAs primarily detect responses to RD1-encoded antigens (e.g., ESAT-6 and CFP-10), which are absent from BCG strains. Contrast-enhanced computed tomography in April 2024 showed an abdominal aortic aneurysm (Figure 1A). Repeat CT and ^18^F-fluorodeoxyglucose positron emission tomography/CT (FDG-PET/CT) in August 2024 demonstrated rapid aneurysm enlargement with increased enhancement and marked FDG uptake in the aortic wall, as well as diffuse bilateral ground-glass opacities (GGOs) in the lungs (Figure 1B–D). Blood cultures obtained on day 0 were negative for common bacteria. After empirical treatment with meropenem and vancomycin that failed to improve his condition, the patient underwent stent graft removal and artificial vessel replacement on day 12. Intraoperatively, an aortic wall incision revealed a large amount of pus, and Ziehl–Neelsen staining revealed acid-fast bacilli. Real-time PCR performed on the pus specimen using the GENECUBE^®^ system (TOYOBO, Osaka, Japan) was positive for the MTBC, and subsequent differential PCR suggested *M. bovis* BCG (see Section 4, Materials and Methods) [20]. The aorta tissue was fixed in formalin and then embedded in paraffin for histopathological studies. The pathology of the surgically removed aortic wall is illustrated in Figure 2. Postoperatively, the GGOs in the lung fields rapidly worsened, and he developed ARDS within 2 weeks after surgery. Acute hypoxemic respiratory failure in this patient meets the Berlin definition of ARDS. On Day 18, the patient developed acute worsening hypoxemia requiring endotracheal intubation. Arterial blood gas analysis on a fraction of inspired oxygen (FiO_2_) of 0.55 demonstrated an arterial partial pressure of oxygen (PaO_2_) of 68.0 mmHg (PaO_2_/FiO_2_ ratio 123.6) under continuous positive airway pressure (CPAP) with positive end-expiratory pressure (PEEP) of 6 cmH_2_O, consistent with moderate ARDS. Systemic corticosteroids were administered for ARDS, consisting of hydrocortisone 200 mg/day for 4 days, followed by methylprednisolone 60 mg/day for 7 days, and then switched to betamethasone 9 mg/day, which was continued thereafter. CT showed traction bronchiectasis, and follow-up CT after corticosteroid initiation demonstrated gradual improvement in ARDS-related changes. Mycobacterial cultures ultimately yielded *M. bovis* from blood drawn on day 0, intraoperative aortic pus obtained on day 12, and BAL fluid obtained on day 18. Aortic tissue and blood culture isolates were subjected to WGS. Isolates were classified as *M. bovis* BCG (TB-Profiler sublineage La1.2.BCG; in silico spoligotype 676773777777600). Genome coverage was >96% (QUAST genome fraction 96.19–96.29%), with high read mapping (99.3%) and median target depths of 79× and 106× (Table S1). The only resistance-associated variant detected was pncA p.His57Asp, consistent with pyrazinamide resistance in *M. bovis* BCG; no resistance-conferring mutations were detected for rifampicin, isoniazid, or ethambutol. No additional known virulence-associated mutations, including those in *esxM*, were identified. Direct mycobacterial PCR on BAL fluid and urine was negative; however, mycobacterial culture of BAL fluid and urine became positive, and subsequent PCR-based differentiation of the culture isolate supported identification as *M. bovis* BCG. Based on these findings, the patient was diagnosed with a mycotic aneurysm and disseminated BCG infection with septic shock and ARDS requiring vasopressors. Antituberculosis therapy with isoniazid (INH; 250 mg/day), rifampicin (RFP; 450 mg/day), and ethambutol (EB; 750 mg/day) was initiated on postoperative day 1, with a planned total treatment duration of 9 months (Figure S2).

## 3. Discussion

This case illustrates a rare but life-threatening spectrum of iBCG-L/SCs—delayed onset disseminated BCG infection presenting with an abdominal mycotic aneurysm and subsequent septic shock and ARDS. Although severe iBCG-L/SCs are uncommon, pooled contemporary analyses indicate a non-negligible case fatality rate among severe presentations, with particularly high mortality reported for disseminated (18.9%), vascular involvement (12.5%), and pulmonary lesions (8.3%) [10]. The present patient’s clinical course—approximately one year after the final iBCG instillation, rapid aneurysm enlargement with intense periaortic FDG uptake, microbiologic confirmation of *M. bovis* BCG in multiple compartments, and progression to ARDS—therefore represents an extreme phenotype that is nonetheless clinically instructive for long-term post-iBCG vigilance.

The pathogenesis of mycotic aneurysms is thought to involve BCG-infected immune cells seeding and weakening the vessel wall [12,13]. In our patient, the presence of an atherosclerotic abdominal aortic aneurysm and intense periaortic FDG uptake support hematogenous and perivascular seeding as plausible routes. After EVAR, the endograft and perigraft remodeling may permit persistence or reseeding [10,12,13].

Source control is central for vascular disease in patients with aortic or large-vessel involvement [10,12,13]. According to a pooled analysis, combined anti-tuberculous therapy plus surgery was associated with markedly lower mortality than other management strategies (3.6% vs. 73%) [10]. This framework aligns with the present case: the patient did not improve with broad-spectrum antibacterial therapy, while intraoperative findings (purulence and acid-fast bacilli) prompted targeted antituberculous therapy and definitive explantation/replacement of infected vascular material, after which systemic status gradually stabilized. These features reinforce a practical point emphasized across case series—early multidisciplinary management involving infectious diseases, vascular surgery, and critical care is often decisive in severe vascular presentations [10,12,13].

The respiratory findings required careful differential diagnosis because pulmonary complications after intravesical BCG therapy may reflect infection, immune-mediated pneumonitis, postoperative factors, or unrelated disease. In this case, diffuse bilateral GGOs were already present on preoperative CT, arguing against surgery as the sole trigger. Bacterial pneumonia was less supported because blood cultures were negative and sputum culture yielded only common oral flora; moreover, routine bacterial cultures of BAL fluid did not detect common bacteria. BAL showed a lymphocyte-predominant profile (WBC 200/µL; lymphocytes 72%) with negative cytology (Class II), and serologic screening for autoimmune-associated interstitial lung disease was negative. Importantly, mycobacterial PCR on BAL was negative, but mycobacterial culture of BAL fluid grew *M. bovis* BCG; PCR performed on the culture isolate subsequently confirmed BCG; together with BCG confirmed from vascular tissue and blood, these findings are most consistent with disseminated BCG disease with pulmonary involvement contributing to progressive respiratory failure and ARDS, potentially with an immune-mediated component.

This case also highlights diagnostic considerations relevant to late-onset iBCG complications. Routine bacterial blood cultures are frequently negative, and microbiologic confirmation often requires tissue sampling with AFB staining, mycobacterial culture, and/or NAAT/PCR [9,10]. Standard IGRAs may be negative in BCG infection because they target RD1-encoded antigens (ESAT-6/CFP-10) that are deleted in BCG. Here, Ziehl–Neelsen staining and real-time PCR of purulent aortic material provided early evidence of a mycobacterial etiology despite negative routine cultures, and subsequent cultures from pus, blood, and bronchoalveolar lavage supported disseminated infection. Because *M. bovis* BCG belongs to the MTBC, species-level identification has therapeutic implications (e.g., intrinsic pyrazinamide resistance) and may not be achievable with non-specific assays [10]. Increasingly, sequencing-based approaches provide higher resolution; WGS has already been applied in selected delayed iBCG-associated prosthetic infections to confirm BCG and support antimycobacterial regimen selection [17].

A central mechanistic question in unusually severe iBCG phenotypes is whether pathogen microevolution contributes to dissemination and organ tropism. BCG substrains used worldwide are genomically diverse [16], and strain-level differences have been proposed to influence immunogenicity and possibly toxicity [18]. Experimental work also implicates specific mycobacterial effectors in dissemination; for example, EsxM (a type VII secretion system–associated effector) has been linked to enhanced dissemination via altered macrophage motility [19]. In our case, WGS of the *M. bovis* BCG clinical isolates demonstrated high concordance with the standard vaccine strain and revealed no drug resistance mutations or known virulence-enhancing mutations, including in *esxM*. These findings make within-host acquisition of established virulence-enhancing variants (including changes in *esxM*) less likely as a dominant driver of the unusually severe vascular and pulmonary phenotype. Accordingly, the case may be better explained by variation in host susceptibility and permissive tissue context.

Host factors remain a major—and incompletely characterized—determinant of both therapeutic response and adverse outcomes after iBCG. Emerging immunogenetic data suggest that HLA and NK-receptor ligand backgrounds modulate clinical outcomes after BCG immunotherapy: HLA-A*11, HLA-B*07, and HLA-B*18 allotypes have been associated with more favorable oncologic outcomes, whereas HLA-B*44 and other KIR3DL1–Bw4 ligand combinations have been associated with unfavorable outcomes [21]. A limitation of the present case is the absence of host immunogenetic susceptibility testing. Whether similar immunogenetic features modulate the risk of disseminated infection remains unclear and warrants a prospective study in severe iBCG complications.

The tissue microenvironment in which BCG persists and disseminates may shape clinical manifestations. Although we did not directly assess bacterial metabolic activity in this patient, disseminated BCG infection has been reported to involve multiple organs, including vascular structures and lungs [10]. These sites may provide lipid-rich, macrophage-infiltrated inflammatory niches, raising the hypothesis that host lipid availability could facilitate intracellular persistence of MTBC organisms, including *M. bovis* BCG. Atherosclerotic arterial walls contain lipid-laden plaques [22], and MTBC organisms can utilize host cholesterol and fatty acids during infection [23]. Taken together, these established features support a hypothesis-generating model in which lipid-rich inflammatory lesions—such as the atherosclerotic infrarenal aneurysm with marked periaortic FDG uptake in our patient—could provide a permissive niche for prolonged persistence of BCG and contribute to delayed vascular infection. This interpretation remains speculative and should be tested in mechanistic studies.

Finally, the long interval between iBCG exposure and overt disease in this case is consistent with latency and reactivation paradigms described for MTBC infections. First, MTBC organisms can enter non-replicating persistence within macrophages and later resume replication; BCG, as an attenuated *M. bovis* lineage, may share components of these persistence programs, although direct evidence specifically demonstrating post-iBCG dormancy/reactivation in human macrophages is limited [24,25]. Experimental models have demonstrated non-replicative persistence and delayed reactivation of *M. bovis*, consistent with latency-like behavior [24,25]. Second, clinical observations indicate that viable *M. bovis* BCG can persist in the urinary tract and may be detectable in urine for months after iBCG, in some cases extending beyond one year after completion of therapy [26]. This supports a “slow-burn” infection model in which subclinical persistence at mucosal or vascular sites precedes overt BCGitis or hematogenous dissemination. The approximately one-year interval between the last iBCG instillation and the onset of systemic symptoms in our patient is consistent with these latency mechanisms and suggests that long-lived intracellular reservoirs and ongoing low-grade antigen exposure may together predispose to late, immunologically driven disease. Dormancy, immune-mediated reactivation, and slow-burning persistence are central paradigms in *M. tuberculosis* pathogenesis, and our observations in disseminated BCG infection are consistent with these broader mechanisms in MTBC disease.

## 4. Materials and Methods

The aortic tissue and blood culture samples were sent to the Tuberculosis Research Institute of the Japan Anti-Tuberculosis Association. DNA was obtained using phenol-chloroform extraction, and the concentration was quantified using a Qubit fluorometer (Life Technologies Holdings Pte Ltd., Singapore). A WGS library was constructed using the QIAseq FX DNA library kit (Qiagen, Hilden, Germany). Sequencing was performed on an Illumina NextSeq 550 platform (Illumina Inc., San Diego, CA, USA) using a NextSeq reagent kit (300 cycles, Illumina Inc., San Diego, CA, USA) with 150-mer paired-end short reads. To profile using WGS, drug resistance profiles and lineage classifications were predicted using TB-Profiler (v2.0).

In the author’s hospital, colonies grown on 2% Ogawa medium were suspended in purified water, heat-treated at 95 °C for 10 min, diluted 1:100 in GENECUBE sample dissolution/lysis solution, and loaded onto the GENECUBE system per the manufacturer’s instructions. Real-time PCR using the GENECUBE^®^ system was also performed directly on the intraoperative purulent aortic material (prior to culture). Briefly, the specimen was mechanically homogenized, heat-treated, and processed with the GENECUBE sample dissolution/lysis solution according to the manufacturer’s instructions before loading onto the GENECUBE system; MTBC detection and subsequent BCG discrimination were interpreted using the same criteria described above. Discrimination of *M. bovis* BCG vs. *M. tuberculosis* was based on a DnaJ1 SNP and QProbe melting curve analysis. PCR was performed on GENECUBE (94 °C 30 s; 50 cycles of 97 °C 1 s, 58 °C 3 s, 63 °C 5 s), followed by automated melting curve acquisition/interpretation. *M. tuberculosis* was defined as a melting peak at 52.0–57.5 °C with a cutoff ≥ 5; BCG as 58.0–66.0 °C with a cutoff ≥ 5. Other results were considered indeterminate and retested from pretreatment. An internal control was co-amplified; failure to detect it was reported as “Invalid”, and the test was repeated [27].

## 5. Conclusions

This case illustrates that serious systemic complications of iBCG can occur even in the absence of identifiable in vivo–acquired, virulence-enhancing mutations in the infecting BCG strain. Our findings underscore the need for prolonged vigilance for late-onset vascular and pulmonary complications after iBCG and highlight the importance of early multidisciplinary management involving infectious disease specialists, vascular surgeons, and intensivists when disseminated BCG infection is suspected. To our knowledge, this is the first report of WGS of a clinical *M. bovis* BCG isolate causing mycotic aneurysm and ARDS.

## Figures and Tables

**Table 1:** Laboratory findings on admission (Day 0).

Parameter	Value	Reference Range	Unit
Inflammation			
C-reactive protein (CRP)	19.42	0.00–0.14	mg/dL
Hematology			
White blood cell count	5.1	3.3–8.6	×10^3^/µL
Hemoglobin	12.0	13.7–16.8	g/dL
Mean corpuscular volume (MCV)	91	83.6–98.2	fL
Platelets	148	158–348	×10^3^/µL
Differential (percent)			
Neutrophils	93.1	39.8–70.5	%
Lymphocytes	4.7	23.1–49.9	%
Monocytes	2.0	4.3–10.0	%
Eosinophils	0.0	0.6–5.4	%
Basophils	0.2	0.3–1.4	%
Liver/biliary enzymes and proteins			
Aspartate aminotransferase (AST)	268	13–30	U/L
Alanine aminotransferase (ALT)	285	10–42	U/L
Alkaline phosphatase (ALP)	580	38–113	U/L
Gamma-glutamyl transferase (GGT)	254	13–64	U/L
Total bilirubin	1.72	0.4–1.5	mg/dL
Total protein	5.6	6.6–8.1	g/dL
Albumin	2.2	4.1–5.1	g/dL
Renal function/electrolytes			
Blood urea nitrogen (BUN)	51.6	8.0–20.0	mg/dL
Creatinine	2.47	0.65–1.07	mg/dL
eGFR	21.14	≥60	mL/min/1.73 m^2^
Sodium	143	138–145	mmol/L
Potassium	4.7	3.6–4.8	mmol/L
Muscle/cardiac markers			
Creatine kinase (CK)	246	59–248	U/L

## Data Availability

The data generated during this study can be provided by the corresponding author upon reasonable request.

## Supplementary Materials

The following supporting information can be downloaded at: https://www.mdpi.com/article/10.3390/ijms27010238/s1.

## Abbreviations

The following abbreviations are used in this manuscript:

AFB	acid-fast bacilli
ARDS	acute respiratory distress syndrome
BAL	bronchoalveolar lavage
BCG	Bacillus Calmette–Guérin
CT	computed tomography
ERCP	endoscopic retrograde cholangiopancreatography
EVAR	endovascular aneurysm repair
FDG-PET/CT	^18^F-fluorodeoxyglucose-positron emission tomography/computed tomography
GGOs	ground-glass opacities
HLA	human leukocyte antigen
iBCG	intravesical Bacillus Calmette–Guérin
iBCG-L/SCs	iBCG-related local/systemic complications
iBCG-ST/SLAEs	iBCG-related short-term/self-limiting adverse events
IGRA	interferon-gamma release assay
NAATs	nucleic acid amplification tests
NMIBC	non-muscle-invasive bladder cancer
MTBC	*Mycobacterium tuberculosis* complex
PCR	polymerase chain reaction
TURBT	transurethral resection of bladder tumor
WGS	whole-genome sequencing
