# Supplementary material for: Whole Genome Sequencing of Clinical Mycobacterium bovis BCG in Disseminated Infection with Mycotic Aneurysm and ARDS After Intravesical Therapy: A Case Report
Source: Int J Mol Sci. 2025 Dec 25;27(1):238. doi: 10.3390/ijms27010238 (PMC12785506; doi:10.3390/ijms27010238)
Supplement: Supplementary file 1 [file ijms-27-00238-s001.zip › Supplementary_Figures_S1-S2_Timelines.pdf]

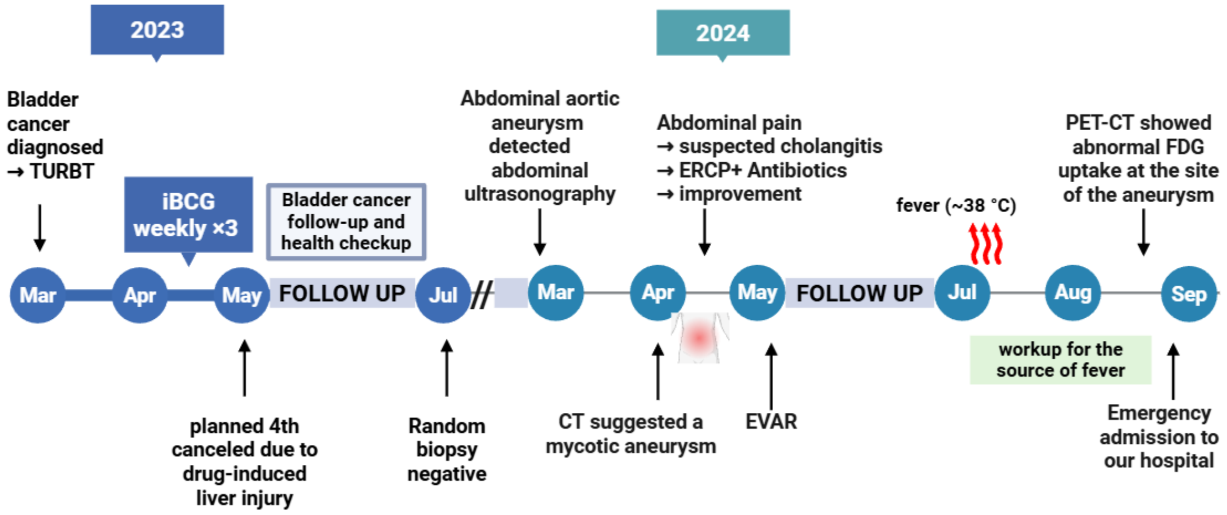

Figure S1. Clinical timeline from intravesical BCG therapy to the diagnosis of suspected infectious abdominal aortic aneurysm and subsequent clinical deterioration.
The patient underwent TURBT for bladder cancer (March 2023) and received intravesical BCG (Immunobladder®, Tokyo-172) weekly for three instillations in April–May 2023; the planned fourth instillation was discontinued because of suspected drug-induced liver injury. Random bladder biopsy in July 2023 showed no recurrence. In March 2024, screening ultrasonography detected an irregular infrarenal abdominal aortic aneurysm; contrast-enhanced CT in April 2024 suggested a possible mycotic aneurysm (partially saccular morphology with irregular mural changes and periaortic fat stranding). The patient underwent ERCP for suspected cholangitis (April 2024) and EVAR (May 2024). Low-grade fever began in July 2024, and FDG-PET/CT in August 2024 demonstrated increased uptake at the aneurysmal wall, followed by emergency admission in mid-August 2024 (Day 0).

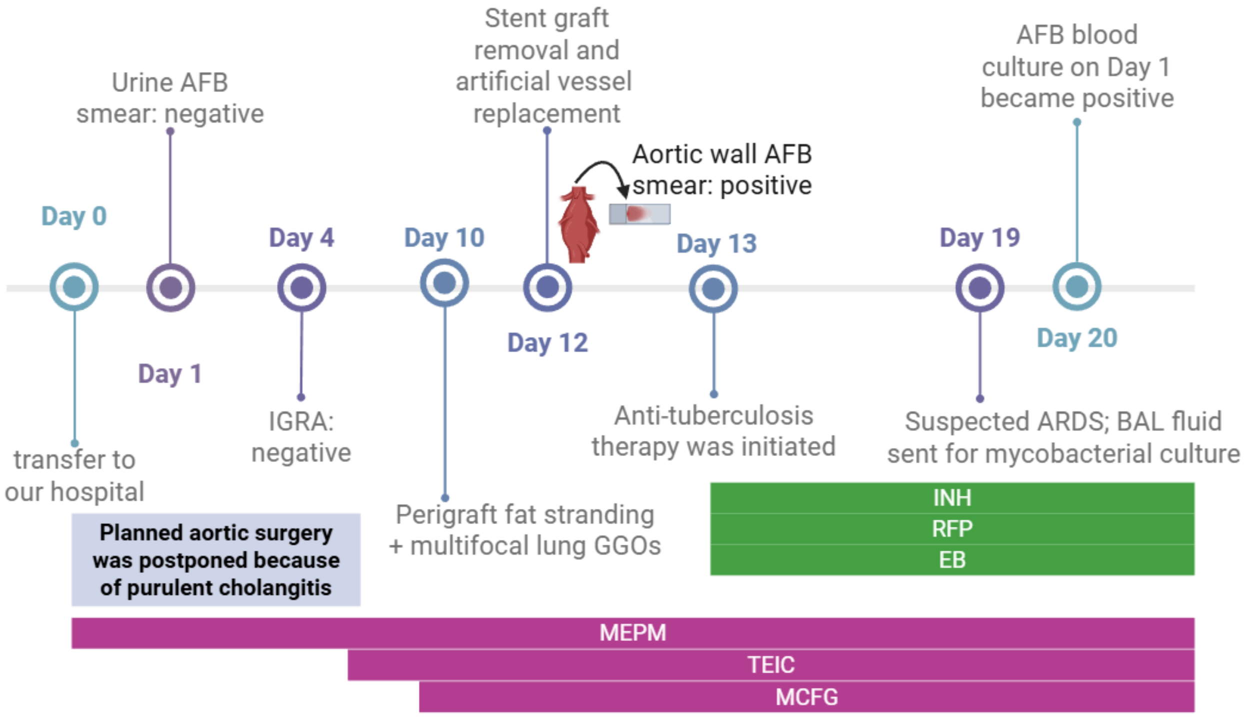

Supplementary Figure S2. Post-admission timeline (Day 0 onward). In-hospital course after emergency admission, showing key investigations, major intervention, microbiology, initiation of anti-tuberculosis therapy, evaluation for ARDS with BAL sampling, and concurrent antimicrobial treatments. **Abbreviations:** AFB, acid-fast bacilli; IGRA, interferon-gamma release assay; ARDS, acute respiratory distress syndrome; BAL, bronchoalveolar lavage; INH, isoniazid; RFP, rifampicin; EB, ethambutol; GGOs, ground-glass opacities; MEPM, meropenem; TEIC, teicoplanin; MCFG, micafungin.
